# Effect of *Lactobacillus sakei* OK67 in Reducing Body and Visceral Fat in Lifestyle-Modified Overweight Individuals: A 12-Week, Randomized, Double-Blind, Placebo-Controlled Clinical Trial

**DOI:** 10.3390/nu15133074

**Published:** 2023-07-07

**Authors:** Seong-Jun Oh, Young-Gyu Cho, Dong-Hyun Kim, Yun-Ha Hwang

**Affiliations:** 1 DONGWHA Pharm Research Institute, 35-71, Topsil-ro, Giheung-gu, Yongin-si 17084, Republic of Korea; seongjun.oh@dong-wha.co.kr; 2 Department of Family Medicine, Seoul Paik Hospital, College of Medicine, Inje University, 9, Mareunnae-ro, Jung-gu, Seoul 04551, Republic of Korea; jacobel@paik.ac.kr; 3 Department of Life and Nanopharmaceutical Sciences, College of Pharmacy, Kyung Hee University, 26, Kyungheedae-ro, Dongdaemun-gu, Seoul 02447, Republic of Korea; dhkim@khu.ac.kr

**Keywords:** *Lactobacillus sakei* OK67 (DW2010), overweight, probiotics, visceral fat

## Abstract

Obesity is a global health problem that affects the quality of life. It is a multidimensional chronic risk factor for major medical conditions, such as cardiovascular diseases, diabetes, and cancer. This clinical trial evaluated the efficacy of *Lactobacillus sakei* OK67 (DW2010), a lactic acid bacterium, in reducing body and visceral fat in overweight individuals (body mass index ≥25 kg/m^2^ and <30 kg/m^2^), aged 20–60 years. A total of 100 subjects placed in a lifestyle modification program were randomly assigned to receive either DW2010 (2.0 g/day, 1.0 × 10^10^ CFU) or a placebo for 12 weeks. The efficacy of DW2010 was evaluated by measuring body fat mass using dual-energy X-ray absorptiometry and visceral fat area using computed tomography. After 12 weeks, the change in body fat in the DW2010 group was not markedly different from that in the placebo group. However, visceral fat area decreased more in the DW2010 group than in the placebo group (*p* = 0.035). During the clinical trial, no major adverse events were reported. Moreover, no statistical differences were observed in the biochemical parameters of the DW2010 and placebo groups. Overall, we concluded that the intake of DW2010 for 12 weeks is safe and potentially reduces visceral fat in lifestyle-modified overweight subjects.

## 1. Introduction

Excessive fat accumulation occurs when calorie consumption exceeds energy expenditure. Specifically, obesity refers to excessive fat accumulation in adipose tissue, thereby leading to low-level inflammation, which in turn induces obesity [1]. Furthermore, fat accumulation around the abdominal viscera is considered to have greater negative health implications than obesity in general [2]. Moreover, abdominal obesity is a major risk factor for systemic inflammation, hyperlipidemia, insulin resistance, and cardiovascular disease [3]. Importantly, abdominal obesity aggravates insulin resistance and metabolic syndrome [4]. Abdominal obesity does not always occur in individuals with an elevated BMI, and individuals of normal weight can be metabolically obese if they have excessive visceral fat [5].

Previous investigations on the effects of high-fat diet (HFD)-induced obesity in humans and mice have demonstrated an increase in Firmicutes and a reduction in Bacteroidetes in the intestinal microflora (gut microbiota) [6]. Upon HFD consumption, the gut microbiota increase the production of lipopolysaccharide, a potent endotoxin found in the outer membrane of Gram-negative bacteria [7,8]. Lipopolysaccharides stimulate the host immune system via the Toll-like receptor 4 and associated mitogen-activated protein kinase signaling system, thereby activating the production of inflammatory cytokines, such as tumor necrosis factor-α and interleukin-1 beta, which further induce inflammatory responses [9]. In addition, lipopolysaccharides reduce the expression of intestinal tight-junction proteins, thereby increasing the absorption of lipopolysaccharides into the bloodstream. Lipopolysaccharides in the bloodstream stimulate the secretion of cytokines in immune cells, thereby leading to lipogenesis in adipose tissue [10].

Live lactic acid bacteria, which are Gram-positive bacteria that show acid resistance, are considered as typical beneficial bacteria in the gut. They are mainly found in yogurt, kimchi, and cheese, and they inhabit the female reproductive organs and intestines of humans and animals [11,12]. Among the beneficial bacteria, several *Lactobacillus* species are considered safe. Moreover, *Lactobacillus* isolated and identified from kimchi regulate host physiology [13,14,15] and improve diabetes by lowering blood sugar levels [16,17]. Specifically, *Lactobacillus sakei* OK67 was found to reduce the HFD-induced lipopolysaccharide levels in the intestine of mice. Furthermore, the oral administration of *L. sakei* OK67 suppressed fasting blood glucose, hyperglycemia, body weight, body fat, epididymal fat, and insulin levels in HFD-treated mice [18].

Therefore, in this study, a randomized, double-blind, placebo-controlled clinical trial was conducted to assess the effect of *L. sakei* OK67 in regulating body and visceral fat in lifestyle-modified overweight individuals.

## 2. Materials and Methods

### 2.1. Study Product

*Lactobacillus sakei* OK67 was manufactured by Kebijen Co., Ltd., Jeonju, Republic of Korea. The *L. sakei* OK67 product used in this clinical trial (DW2010) had the appearance of a milk-white powder. The placebo used in this study was made of a dextrin mixture and had a similar appearance, flavor, and weight as DW2010. Subjects ingested DW2010 or a placebo every day from June 2018 to November 2019 (2.0 g/day, 1.0 × 10^10^ CFU). The product used in this clinical trial was stored at temperatures between 0–10 °C.

### 2.2. Subjects

The clinical trial was conducted from June 2018 to November 2019 at Inje University Seoul Paik Hospital in compliance with the Helsinki Declaration and Korean Good Clinical Practice guidelines. The clinical trial was approved by the Institutional Review Board (IRB No. PAIK 2018-04-004), and registered at the Clinical Research Information Service of South Korea (Approval Number: KCT0003222). A total of 100 subjects were recruited after screening (physical and laboratory examinations). All subjects provided informed consent prior to the initiation of the clinical trial. The following selection criteria were used: (1) male and female subjects aged ≥20 and ≤60 years old; (2) those with a BMI of ≥25 kg/m^2^ and <30 kg/m^2^; (3) those who had not consumed alcohol within a month and could abstain from alcohol during the study period; and (4) those who consented to participate in this clinical trial and signed informed consent before the study initiation.

The exclusion criteria for clinical trial subjects were: (1) those who suffered from or were diagnosed with severe cerebrovascular disease (including cerebral infarction and hemorrhage) or heart disease (angina pectoris, myocardial infarction, heart failure, and arrhythmia) within the last six months; (2) patients with uncontrolled hypertension (blood pressure ≥ 160/100 mmHg); (3) persons with a fasting blood glucose level of ≥126 mg/dL, a random blood glucose level of ≥200 mg/dL, or a diabetic patient taking oral hypoglycemic agents or insulin; (4) persons with thyroid stimulating hormone levels of ≤0.1 µU/mL or ≥10 µU/mL; (5) those whose creatinine level was more than twice the normal upper limit; (6) those whose aspartate aminotransferase (AST) and alanine aminotransferase (ALT) levels were more than three times the normal upper limit; (7) those who complained of severe gastrointestinal symptoms, such as heartburn and indigestion; (8) those who had been taking drugs that affect body weight (absorption inhibitors and appetite suppressants, supplements related to obesity improvement, psychiatric drugs, beta-blockers, diuretics, contraceptives, steroids, or female hormones) within the last month; (9) those who had continuously consumed probiotics (fermented milk) within the last week; (10) those who had lost ≥3 kg within four weeks of screening; (11) those who had participated in a commercial obesity program within the last three months; (12) those who had participated or planned to participate in other clinical studies within the month before clinical screening; (13) those who had been hospitalized or were undergoing drug treatment or rehabilitation treatment for disorders induced by alcohol use, heart disease, or central nervous system disorders identified through a medical history survey; (14) those who were deemed unable to exercise due to musculoskeletal disorders; (15) those who were pregnant or planning to become pregnant during this clinical study or lactating women; and (16) those deemed ineligible by the clinical trial manager for other reasons.

### 2.3. Study Design

The clinical trial was a 12-week, double-blind, randomized controlled trial to assess the efficacy and safety of DW2010 in regulating body and visceral fat in overweight individuals for weight loss purposes. After receiving written consent, subjects were randomly assigned to either the DW2010 or placebo group (*n* = 50/group). During the intervention period, all subjects were instructed not to take any dietary supplements or traditional medicines, engage in strenuous physical activity, or consume alcohol. Compliance with these regulations were monitored at each visit. Moreover, subjects were instructed to report any adverse events (AEs) or changes to their routine exercising, lifestyle, food habits, or other relevant habits. We referred to the paper evaluating body fat mass, which detailed the same major evaluation variables in this clinical study. According to the study by Nosaka et al. [19], the fat mass changed by −4.2 ± 2.8 kg from the baseline and decreased by 1.3 ± 0.8 kg compared to the placebo group. Considering this result, we calculated a sample size of 50 subjects per group, assuming a statistical power of 0.80, a two-tailed significance level of 0.05 and a dropout rate of 30%. After randomization into the DW2010 and placebo groups in a 1:1 ratio, the clinical trial was conducted for 12 weeks on 74 subjects, excluding dropouts.

### 2.4. Diet and Physical Activity Counseling

During the clinical trial, all subjects from both groups were instructed to reduce energy intake by 500 kcal/day. All subjects were instructed to burn 300 kcal or more daily by working, leisure, and moving, including physical activity. Dietary intake and physical activity were evaluated at an interim and last visit by means of meal and physical activity sheets. The average daily energy intake was analyzed using CAN-Pro 5.0, which is a software developed for nutritional evaluation considering the type, amount, and number of dietary intake, and the average daily calories consumed were analyzed using the Metabolic Equivalent for Task (MET) according to the type, time, and amount of exercise recorded in the physical activity sheets for at least 3 days (including 1 weekend day). 

There was no statistically significant difference in energy intake and physical activity between the DW2010 group and the placebo group after 12 weeks (Table 1).

### 2.5. Efficacy Measurements

The primary outcome was change in body fat (g). Body fat mass, body fat percentage, and lean body mass were measured using dual-energy X-ray absorptiometry (DEXA; Prodigy^®^ DEXA Lunar, GE Healthcare, Madison, WI, USA) at baseline (0 weeks) and 12 weeks. Changes in visceral fat, the secondary outcome, were assessed using computed tomography (CT), which could accurately determine the area of visceral fat in the abdomen. The area of visceral fat and subcutaneous fat of the abdomen was measured by the cross section between the fourth and fifth lumbar vertebrae. It was measured at baseline (0 weeks) and 12 weeks. 

### 2.6. Safety Measurements

At each visit, subjects underwent routine biochemical assessments and measurements, including AST, ALT, albumin, alkaline phosphatase (ALP), creatine, *blood urea nitrogen* (BUN), uric acid, glucose, total bilirubin, γ-glutamyl transpeptidase (GTP), systolic and diastolic blood pressure, and pulse tests. Blood parameters were measured using XN-3000, UF-5000 (Sysmex, Norderstedt, Germany), and AU5800 (Beckman Coulter, CA, USA) blood analyzers. Furthermore, AEs were recorded and listed to determine their frequency and severity.

### 2.7. Statistical Analysis

Statistical analysis was performed using SAS^®^ v. 9.4 (SAS Institute, Cary, NC, USA). A two-sample *t*-test, Wilcoxon rank sum test, chi-square test, or Fisher’s exact test was performed for demographic, efficacy, and safety evaluation. In addition, changes from the baseline in each parameter between the two groups were analyzed using analysis of covariance. The data obtained from this clinical trial were presented by calculating the mean and standard deviation with appropriate technical statistics, and the significance of the difference was verified at a *p*-value of <0.05 level with two-sided tests. All *p*-values are reported using four digits.

## 3. Results

### 3.1. Subject Baseline Characteristics

**Figure 1:**
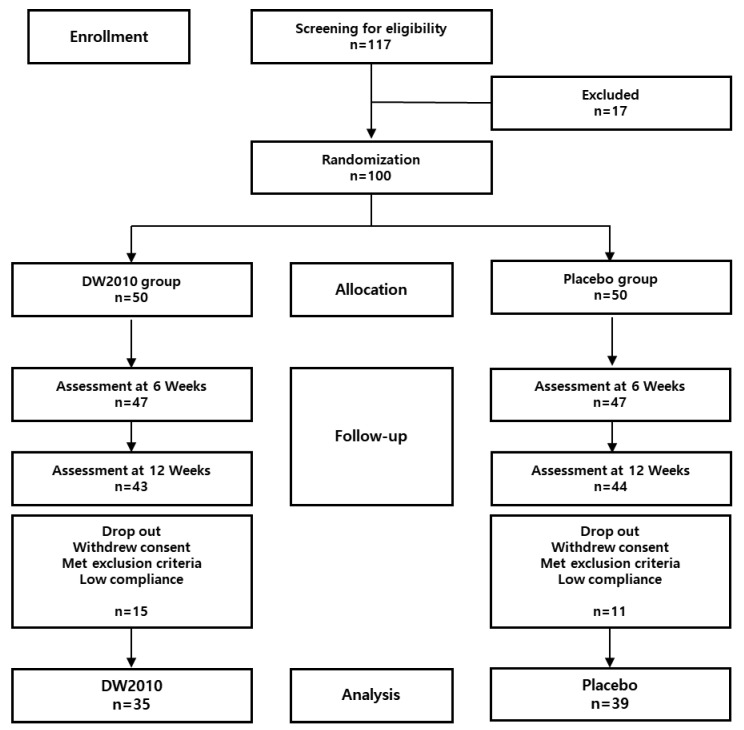
Flow diagram showing the selection and distribution of subjects in the clinical trial of the effects of *Lactobacillus sakei* OK67 (DW2010) on body and visceral fat reduction in overweight individuals.

In total, 117 volunteers were initially selected to participate in this clinical trial, of whom 17 were excluded during screening. Therefore, the DW2010 and placebo groups comprised 50 subjects each. However, during the investigation, 15 subjects from the DW2010 group who withdrew consent or met exclusion criteria, or who exhibited low trial compliance (≤65%), were excluded from the clinical trial. Similarly, 11 subjects from the placebo group were removed from the assessment. Therefore, 74 subjects (DW2010 group, *n* = 35; placebo group, *n* = 39) were included in the final analysis (Figure 1). In the DW2010 group, the proportions of males and females were 31.4% and 68.6%, respectively. In the placebo group, males and females constituted 43.6% and 56.4%, respectively. The average ages in the DW2010 and placebo groups were 39.9 ± 9.7 y and 42.1 ± 1.0 y, respectively. There were no statistically significant differences between the two groups regarding sex distribution, age, or smoking habits. The average compliance levels in the DW2010 and placebo groups were 97.9 ± 9.5% and 96.7 ± 8.2%, respectively. No significant differences were found between the DW2010 and placebo groups in the baseline measurement values for demographics, anthropometry, body composition, laboratory tests, or daily energy intake. There were also no differences in daily energy intake between the two groups at 0 weeks. The general characteristics of the subjects are shown in Table 2.

### 3.2. Primary Outcome Assessment

**Figure 2:**
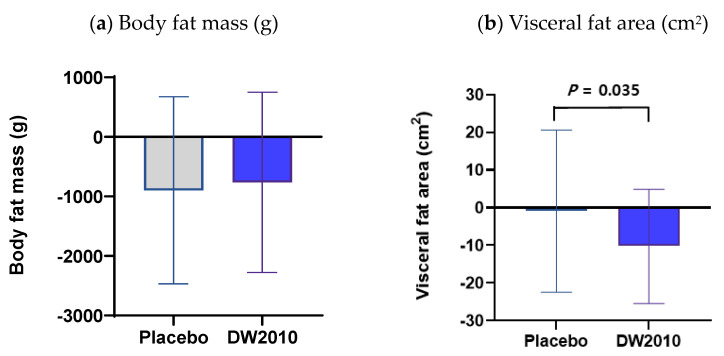
Mean changes to (**a**) body fat mass and (**b**) visceral fat area caused by *Lactobacillus sakei* OK67 (DW2010) after 12 weeks. Negative values correspond to decreases from week 0 to week 12.

DEXA scanning revealed that the DW2010 group subjects lost on average 764.17 ± 1513.1 g of body fat mass after 12 weeks of consumption, while the placebo group subjects lost on average 898.0 ± 1 571.3 g. However, anthropometry and body composition, including body fat mass, did not differ markedly between the groups at 0 or 12 weeks (Table 3, Figure 2).

### 3.3. Secondary Outcome Assessment

CT scanning revealed that the DW2010 group subjects lost on average 10.27 ± 15.17 cm^2^ of visceral fat area after 12 weeks of consumption, while the placebo group subjects lost on average 0.94 ± 21.51 cm^2^. Interestingly, the difference between the groups in visceral fat area after 12 weeks of consumption was statistically significant (*p* = 0.035) (Table 4, Figure 2). However, the subcutaneous fat area did not differ substantially between the groups at 12 weeks.

### 3.4. Safety Outcome Assessment

All safety parameters, including blood pressure, liver function tests, and renal function tests, were within normal ranges from the baseline until the end of the study, with no significant differences found between the DW2010 and placebo groups. To identify potential AEs, the clinical condition of each individual was evaluated at predetermined intervals. During the clinical trial period, no major AEs were reported. There were no noticeable differences in specific parameters between the DW2010 and placebo groups. All major parameters were measured as part of the safety evaluation. Mild AEs occurred in seven cases in the DW2010 group and in four cases in the placebo group. Mild AEs included dermatitis contact, rashes, and abdominal pain in the DW2010 group. However, there was no direct association between these mild AEs and DW2010 ingestion (Table 5). There were no withdrawals from the trial due to serious AEs. The safety parameters and respective analyses are described in Table 6.

## 4. Discussion

This clinical trial examined changes in the body and visceral fat of overweight individuals who received DW2010 for 12 weeks. We observed no appreciable differences in body fat (primary outcome) between the groups. However, the visceral fat (secondary outcome) of the DW2010 group differed substantially from that of the placebo group at 12 weeks. *Lactobacillus sakei* OK67 is a typical Gram-positive, anaerobic, acid-resistant lactic acid bacterium typically found in large amounts in kimchi, which is a traditional fermented food. It inhibits gut microbiota lipopolysaccharide production, and alleviates lipopolysaccharide-induced inflammation in mice [18]. In vitro, kimchi-isolated *L. sakei* proBio65 exhibited immunomodulatory properties by elevating Foxp3 protein expression [20]. Similarly, in a collagen-induced arthritis mouse model, *L. sakei* exerted an anti-inflammatory effect by regulating Th17 and regulatory B-cell differentiation [21]. In addition, *L. sakei* WIKIM30 was found to alleviate atopic dermatitis by regulating intestinal flora and regulatory T cells [22]. *Lactobacillus sakei* ADM14 was found to exert an anti-obesity effect in an obese mouse model by causing changes in the intestinal flora [23].

In mice with HFD-induced obesity, DW2010 induced AMP-activated protein kinase activation in the intestine and liver by inhibiting the gut microbiota and endotoxin production; furthermore, it suppressed body weight, hyperglycemia, epididymal fat, and insulin levels [18]. A previous study of HFD-fed mice reported anti-obesity outcomes related to inflammatory state modulation, lipogenic gene downregulation, and adipocyte number reduction [24]. Epicardial fat and abdominal visceral fat surround the myocardium and gastrointestinal organs, respectively, and both are considered to be visceral fat [25]. These interesting observations in mice are consistent with the outcomes of this study, thereby reflecting the high degree of similarity between animal and human investigations. 

Visceral fat triggers changes in the blood lipid profile, inflammation, and insulin resistance. Moreover, obesity is highly associated with visceral fat, which is recognized as the most direct risk factor for the development of obesity-related metabolic disorders [26,27]. As observed in this clinical trial, visceral fat was considerably regulated upon the ingestion of DW2010. Thus, the substantial reduction in visceral fat may have contributed positively to the regulation of obesity in participants. Dietary modifications, specific dietary supplements, and drugs can modify body fat mass in a very short time, although the effects may be temporary. However, reducing accumulated abdominal visceral fat could be challenging.

## 5. Conclusions

In this clinical trial, visceral fat was substantially reduced in the DW2010 group for 12 weeks, thus indicating the significance of DW2010 in visceral fat reduction. Moreover, visceral fat reduction could reduce the risk factors associated with obesity and metabolic syndrome. DW2010 is clinically safe and has therapeutic potential for treating obesity by regulating visceral fat. Subsequent studies are required to investigate the long-term visceral fat effects of DW2010 on human health.

## Figures and Tables

**Table 1:** Changes in energy intake and physical activity during the clinical trial.

	DW2010 Group (*n* = 35)	Placebo Group (*n* = 39)	*p*-Value
	Mean ± SD	Mean ± SD	
Energy intake (kcal/day)			
0 weeks	1824.71 ± 700.08	1757.08 ± 437.67	0.6248 *
12 weeks	1583.30 ± 432.14	1393.53 ± 399.05	
Change from baseline	−241.40 ± 749.42	−349.56 ± 455.11	0.4653 *
Physical activity (kcal/day)			
0 weeks	290.30 ± 192.82	347.44 ± 261.72	0.3541 *
12 weeks	286.81 ± 225.64	287.45 ± 163.76	
Change from baseline	−3.49 ± 197.77	−59.99 ± 215.94	0.2200 *

* Comparison between the DW2010 and placebo groups; two-sample *t*-test; SD, standard deviation.

**Table 2:** Baseline demographic characteristics and body composition of the subjects.

Variables		DW2010 Group (*n* = 35)	Placebo Group (*n* = 39)	*p*-Value
Sex No. (%)	Male	11 (31.4)	17 (43.6)	0.4476 ^†^
	Female	24 (68.6)	22 (56.4)	
Age (years)	Mean ± SD	39.9 ± 9.7	42.1 ± 10.0	0.3596 *
	Min, Max	21.0, 59.0	24.0, 59.0	
Smoking status No. (%)	Non-smoking	32 (91.4)	36 (92.3)	0.9598 ^‡^
	Ex-Smoker	1 (2.9)	0 (0.00)	
	Smoker	2 (5.7)	3 (7.7)	
Smoking amount	Among smokers, number of cigarettes/day			0.7671 **
	Mean ± SD	10.0 ± 7.1	7.0 ± 5.2	
	Min, Max	5.0, 15.0	1.0, 10.0	
Smoking period	Among smokers, years			0.8215 *
	Mean ± SD	15.0 ± 7.1	17.7 ± 13.7	
	Min, Max	10.0, 20.0	3.0, 30.0	
Weight (kg)	Mean ± SD	74.86 ± 9.30	73.25 ± 10.46	0.7047 **
BMI (kg/m^2^)		27.20 ± 1.53	27.09 ± 1.56	0.7534 **
BFM (g)		26,593 ± 4967	25,281 ± 4743	0.2492 *
LBM (g)		44,983 ± 851	44,917 ± 963	0.8795 **
WC (cm)		92.74 ± 5.86	91.29 ± 7.10	0.3452 *
T-Chol (mg/dL)		205.31 ± 43.25	210.21 ± 36.97	0.6017 **
TG (mg/dL)		131.77 ± 86.30	108.46 ± 56.62	0.2511 **
HDL-C (mg/dL)		50.54 ± 10.51	54.08 ± 9.84	0.1396 **
LDL-C (mg/dL)		128.40 ± 37.53	134.41 ± 33.61	0.4697 **
AST (IU/L)		25.46 ± 7.36	25.74 ± 10.57	0.5829 **
ALT (IU/L)		25.58 ± 14.64	27.00 ± 22.49	0.5669 **
Albumin (g/dL)		4.48 ± 0.29	4.48 ± 0.28	0.9439 *
ALP (IU/L)		59.82 ± 13.07	59.26 ± 13.74	0.8350 *
Creatinine (mg/dL)		0.74 ± 0.16	0.75 ± 0.16	0.4626 **
BUN (mg/dL)		13.02 ± 3.66	12.92 ± 3.19	0.9778 **
Uric acid (mg/dL)		5.53 ± 1.34	5.83 ± 1.45	0.3662 **
γ-GTP (IU/L)		23.04 ± 15.70	28.30 ± 26.83	0.5668 **
SBP (mmHg)		126.28 ± 14.59	125.88 ± 13.53	0.8873 *
DBP (mmHg)		78.62 ± 10.20	77.80 ± 8.02	0.7745 **
Energy intake (Kcal/day)		1824.7 ± 700.1	1757.1 ± 437.7	0.6248 *

* Two-sample *t*-test; ** Wilcoxon rank sum test; ^†^ Chi-square test; ^‡^ Fisher’s exact test; SD: standard deviation; BFM: body fat mass; LBM: lean body mass; WC: waist circumference; BMI: body mass index; T-Chol: total cholesterol; TG: triglyceride; HDL-C: high-density lipoprotein cholesterol; LDL-C: low-density lipoprotein cholesterol; AST: aspartate aminotransferase; ALT: alanine aminotransferase; ALP: alkaline phosphatase; BUN: blood urea nitrogen; GTP: glutamyl transpeptidase; SBP: systolic blood pressure; DBP: diastolic blood pressure.

**Table 3:** Effects of *Lactobacillus sakei* OK67 (DW2010) on body fat mass (g) measured using dual-energy X-ray absorptiometry (DEXA).

	DW2010 Group (*n* = 35)	Placebo Group (*n* = 39)	*p*-Value
	Mean ± SD	Mean ± SD	
Body fat mass (g)			
0 weeks	26,593 ± 4967	25,281 ± 4743	0.2492 *
12 weeks	25,829 ± 4876	24,383 ± 4661	
Change from baseline	−764.17 ± 1513	−897.97 ± 1571	0.5333 ^$^
Body fat percentage (%)			
0 weeks	37.39 ± 6.54	36.44 ± 6.98	0.5523 *
12 weeks	37.09 ± 6.65	35.86 ± 7.06	
Change from baseline	−0.29 ± 1.68	−0.58 ±1.45	0.4200 ^$^
Lean body mass (g)			
0 weeks	44,983 ± 8505	44,917 ± 9626	0.8795 *
12 weeks	44,318 ± 8681	44,402 ± 9426	
Change from baseline	−665.34 ± 1390	−515.46 ± 1140	0.6153 ^$^
Body weight (kg)			
0 weeks	74.86 ± 9.30	73.25 ± 10.46	0.7047 *
12 weeks	73.67 ± 9.30	72.37 ± 10.09	
Change from baseline	−1.19 ± 1.66	−0.88 ± 1.88	0.5333 ^$^
BMI (kg/m^2^)			
0 weeks	27.20 ± 1.53	27.09 ± 1.56	0.7534 **
12 weeks	26.77 ± 1.61	26.79 ± 1.74	
Change from baseline	−0.43 ± 0.61	−0.30 ± 0.69	0.3889 ^$^
WC (cm)			
0 weeks	92.74 ± 5.86	91.29 ± 7.10	0.3452 *
12 weeks	91.64 ± 5.49	90.27 ± 6.97	
Change from baseline	−1.10 ± 2.44	−1.05 ± 2.71	0.8860 ^$^

* Comparing the DW2010 and placebo groups using a two-sample *t*-test; ** Wilcoxon rank sum test; ^$^ Comparing the DW2010 and placebo groups using analysis of covariance with an adjusted baseline.

**Table 4:** Effect of *Lactobacillus sakei* OK67 (DW2010) on visceral fat area (cm^2^) measured using computed tomography (CT).

	DW2010 Group (*n* = 35)	Placebo Group (*n* = 39)	*p*-Value
	Mean ± SD	Mean ± SD	
Visceral fat area (cm^2^)			
0 weeks	119.43 ± 42.99	117.95 ± 49.70	0.6184 **
12 weeks	109.15 ± 44.12	117.01 ± 47.90	
Change from baseline	−10.27 ± 15.17	−0.94 ± 21.51	0.0354 ^$^
Subcutaneous fat area (cm^2^)			
0 weeks	234.37 ± 56.22	225.05 ± 56.47	0.2960 **
12 weeks	219.74 ± 55.54	214.64 ± 53.01	
Change from baseline	−14.63 ± 22.68	−10.41 ± 32.39	0.6683 ^$^

** Comparing the DW2010 and placebo groups using Wilcoxon rank sum test; ^$^ Comparing the DW2010 and placebo groups using analysis of covariance with an adjusted baseline.

**Table 5:** AE occurrences and respective analyses.

		DW2010 Group (*n* = 50)		Placebo Group (*n* = 50)		Total (N = 100)	*p*-Value
	N	Incidence (%)	N	Incidence (%)	N	Incidence (%)	
Mild	7	14	4	8	11	11	-
Moderate	0	0	0	0	0	0	
Severe	0	0	0	0	0	0	
Relationship with the test article	0	0	0	0	0	0	0.9207 ^‡^

^‡^ *p*-value for Fisher’s exact test.

**Table 6:** Safety parameters in the groups ingesting *Lactobacillus sakei* OK67 (DW2010) or the placebo.

	DW2010 Group (*n* = 50)	Placebo Group (*n* = 50)	*p*-Value
	Mean ± SD	Mean ± SD	
AST (GOT) (IU/L)			
0 weeks	25.46 ± 7.36	25.74 ± 10.57	0.5829 **
12 weeks	25.74 ± 8.01	26.95 ± 13.00	0.1385 **
ALT (GPT) (IU/L)			
0 weeks	25.58 ± 14.64	27.00 ± 22.49	0.5669 **
12 weeks	23.77 ± 12.90	27.81 ± 22.40	0.6218 **
Albumin (g/dL)			
0 weeks	4.48 ± 0.29	4.48 ± 0.28	0.9439 *
12 weeks	4.50 ± 0.23	4.54 ± 0.27	0.8415 **
ALP (IU/L)			
0 weeks	59.8 ± 13.1	59.3 ± 13.7	0.8350 *
12 weeks	62.4 ± 15.2	61.2 ± 14.4	0.8052 **
Creatinine (mg/dL)			
0 weeks	0.74 ± 0.16	0.75 ± 0.16	0.4626 **
12 weeks	0.74 ± 0.19	0.76 ± 0.17	0.0768 *
BUN (mg/dL)			
0 weeks	13.02 ± 3.66	12.92 ± 3.19	0.9778 **
12 weeks	12.88 ± 3.77	13.35 ± 3.25	0.9509 *
Uric acid (mg/dL)			
0 weeks	5.53 ± 1.34	5.83 ± 1.45	0.3662 **
12 weeks	5.40 ± 1.30	5.69 ± 1.45	0.1493 *
Glucose (mg/dL)			
0 weeks	95.98 ± 11.68	91.60 ± 9.58	0.2460 *
12 weeks	95.98 ± 17.85	92.16 ± 7.96	0.1900 **
Total Bilirubin (mg/dL)			
0 weeks	0.71 ± 0.27	0.82 ± 0.33	0.0858 **
12 weeks	0.73 ± 0.34	0.77 ± 0.29	0.2027 **
γ-GTP (IU/L)			
0 weeks	23.04 ± 15.70	28.30 ± 26.83	0.5668 **
12 weeks	20.72 ± 14.54	28.14 ± 26.35	0.8181 **
SBP (mmHg)			
0 weeks	126.28 ± 14.59	125.88 ± 13.53	0.8873 *
12 weeks	128.58 ± 12.60	127.57 ± 11.53	0.8574 *
DBP (mmHg)			
0 weeks	78.62 ± 10.20	77.80 ± 8.02	0.7745 **
12 weeks	79.02 ± 9.60	78.30 ± 8.33	0.6530 *

* Comparing the DW2010 and placebo groups using two-sample *t*-test. ** Comparing the DW2010 and placebo groups using Wilcoxon rank sum test. AST: aspartate aminotransferase; ALT: alanine aminotransferase; GOT: glutamic oxaloacetic transaminase; GPT: glutamic pyruvic transaminase; ALP: alkaline phosphatase; BUN: blood urea nitrogen; GTP: glutamyl transpeptidase; SBP: systolic blood pressure; DBP: diastolic blood pressure.

## Data Availability

The datasets used and/or analyzed during the current study are available from the corresponding author upon reasonable request.
